# A pilot study on assessing the gap between nurses’ task performances and knowledge pertaining the same with reference to “ I COUGH” initiative- a call for promoting patient ‘care bundle’ assignments in low-income nations

**DOI:** 10.12688/f1000research.18815.1

**Published:** 2019-04-23

**Authors:** Sunil Munakomi, Sangam Shrestha, Anita Luitel

**Affiliations:** 1 Neurosurgery, Nobel Medical College and Teaching Hospital, Biratnagar, 0977, Nepal; 2 Koshi Zonal Hospital, Pediatrics, Biratnagar, Morang, 0977, Nepal

**Keywords:** Knowledge, task performance, care bundle

## Abstract

**Background: **The health sector in low-income nations has been crippled owing to low resources, lack of trained staff and a scarcity of effective health-related reforms. Amidst such a scenario, implementation of patient-centered care bundle approaches could help reprise the autonomy and standards of care for healthcare providers as well as safeguard patient safety.

**Methods: **We sought to determine the gap between task performance and the underlying knowledge pertaining the same among nurses from intensive and high dependency neurosurgical units within three hospitals in Nepal through a questionnaire-based approach focusing on task assignments to prevent pulmonary complications among their patients and scoring them with references to   the variables of ‘I COUGH’, a similar patient care bundle initiative.

**Results: **There is a gross discrepancy between the patterns of task performance and the knowledge regarding the rationale behind the same tasks among nurses working in critical care neurosurgical units. In reference to I COUGH, nurses had below 50% knowledge on interventions aimed to prevent pulmonary complications among their patients, irrespective of the level of experience attained in the units. Furthermore, none of them had complete knowledge regarding all components of effective chest physiotherapy.

**Conclusion: **There is the utmost need for the implementation of patient-focused care bundle approaches in upraising the health delivery standards, especially in low-income nations. Such initiatives can promote autonomy amongst healthcare professionals on patient care as well as assuring better patient outcomes by minimizing complications.

## Introduction

Among surgical specialties worldwide, there is alarmingly high incidence of post-operative pulmonary complications, being reported as high as 40%, and leading to 30 days post-surgery mortality for almost 20% ^1^. These complications also lead to an increase in average hospital stay by 8 days and thereby increase in average hospital costs by almost 55% ^1^ Therefore, there is a substantial need to produce a care bundle based on an enhanced recovery protocol within these surgical specialties ^1^. 

In context of low-income nations like Nepal, with a per capita income of below US $700, the current health sector situation is even more alarming. The limited resources available coupled with political turmoil further exacerbates the issue ^2^. It reportedly has only 16.7 intensive care unit (ICU) beds per million of its population ^3^. Even in the capital city, only 7.2 ICU beds equipped to provide backup ventilation are available per 100,000 of the population. Moreover, there is a significant shortage of effective manpower, with a nurse patient ratio of >1:4 during night shifts ^4^. The present nurse to population ratio in Nepal is approximately 11 nurses per 10,000 of the population (with 50 considered to be optimum) ^5^. Recruitment of nurses is low in national hospitals with many opting to go abroad owing to higher salaries, improved facilities and relatively better scope for career building opportunity. This accounts for the temporary employment pattern prevalent in our hospitals; with one study showing up to 50 nurses leaving their hospital within a single calendar year. Moreover, lack of in-service education and timely reforms of the existing nursing guidelines have further affected the nursing profession ^5^.

The I COUGH program is a postoperative pulmonary care bundle approach aimed at reducing the incidence of postoperative pneumonia and unplanned intubation among patients ^6^. This acronym incorporates six variables namely 1) Incentive spirometry, 2) Cough/ chest physiotherapy, 3) Oral care, 4) Understanding, 5) Get out of bed and 6) Head end elevation ^6^.

Such initiative not only helped promote patient’s health but also in improving performance standards and thereby their autonomy ^1^. Therefore, currently upmost emphasis is being given for promoting such performance appraisal methods ^1^. There is upmost need for bridging the gap between nurses’ performance and knowledge of the underlying reasons for the tasks they perform through the application of care bundle approaches such as the I COUGH initiative ^6^.

We performed a pilot study to identify significant discrepancy between the knowledge of and performance of relevant tasks among health care providers in the critical care setting up in regards to the I COUGH initiative, and thereby call for initiation of care bundle approaches in our context as well.

## Methods

### Subjects

A questionnaire based observational analytical study was carried out aiming for maximum inclusion of all nurses practicing in the intensive care and the high dependency neurosurgical units from three major teaching hospitals of Kathmandu University namely 1) Nobel Medical College, Biratnagar 2) College of Medical Sciences, Chitwan and 3) National Institute of Neurosurgery and Allied Health Sciences, Kathmandu were enrolled for our study. We initially confirmed the practice of inclusions of all the variables listed within the I COUGH initiative among the daily assigned nursing task assignments in all these three hospitals. We then opted to determine the level of discrepancy between their patterns of work performance with the underlying knowledge governing the same. The authors requested the management team of each institution to make provisions for maximum attendance of their nurses from their intensive and the high dependency care units in our short educational programme. Prior to beginning the educational course, the authors asked all of the participating 101 nurses to list in numerical order major components of their daily tasks aimed at reducing the risk of pulmonary complications among their patients. They were also advised to tabulate different components of effective chest physiotherapy. Their answers were recorded by a researcher, marked, tabulated and scored out of a total score of six with one score allocated for each of the six variables within the already validated I COUGH protocol. In order to reduce possible bias from early knowledge of the test, the authors performed the test in a single group session the same day of obtaining permission from the respective hospital administrations. The answer session was followed by a short educational class conducted by the researchers describing the importance of application of patient care bundle approach measures such as I COUGH in safeguarding their patients’ lives.

### Data acquisition and analysis

The acquisition of data was done for score of each of the six variables of I COUGH program for each of the participating 101 nurses from the three hospitals. The results were analyzed and tabulated with the help of Windows Excel version 2007 for our result analysis. Frequency analysis was performed on data gathered from nurses.

### Ethical considerations

The study was approved by the Institutional Review Committee (IRC) of Nobel Medical College and Teaching Hospital (NMCTH) (approval number 259/2019). Permission for conducting the research was obtained from each of the hospitals that were included in the study.

## Results

**Figure 1.**
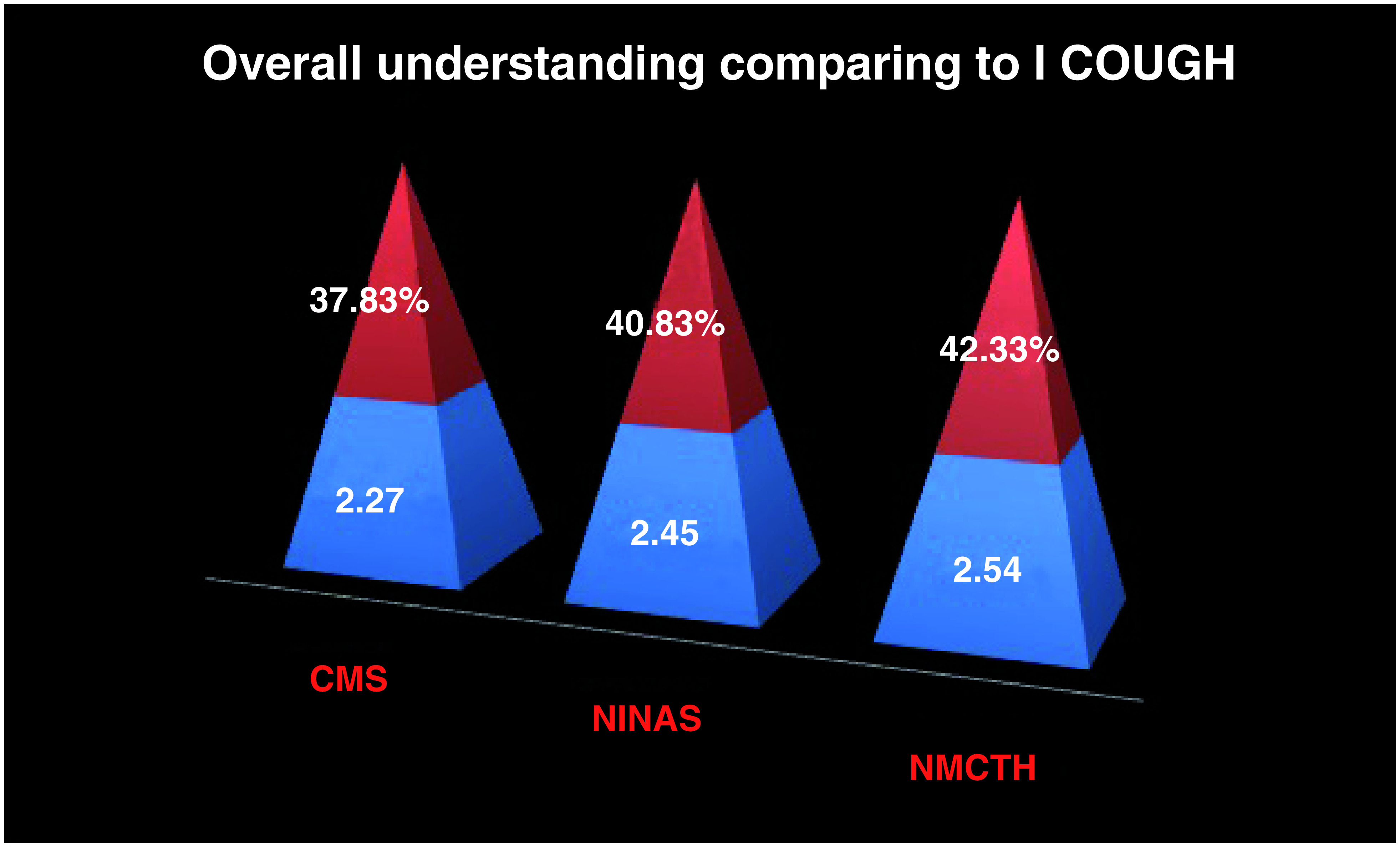
Overall understanding comparing to I COUGH among nurses of different hospitals. NMCTH - Nobel Medical College and Teaching Hospital, CMS - College of Medical Sciences, Chitwan, NINAS - National Institute of Neurosurgery and Allied Health Sciences.

A total of 101 nurses working in the intensive care and high dependency neurosurgical units were enrolled for our study within three different teaching hospitals under Kathmandu University. The average scores obtained by the nurses from three hospitals were below 50% scores (37.83%, 40.83% and 42.33% respectively) in terms of their knowledge while comparing to the I COUGH strategy items ( Figure 1). The average scores of the nurses were 2.27, 2.45 and 2.54 for CMS, NINAS and NMCTH respectively.

**Figure 2.**
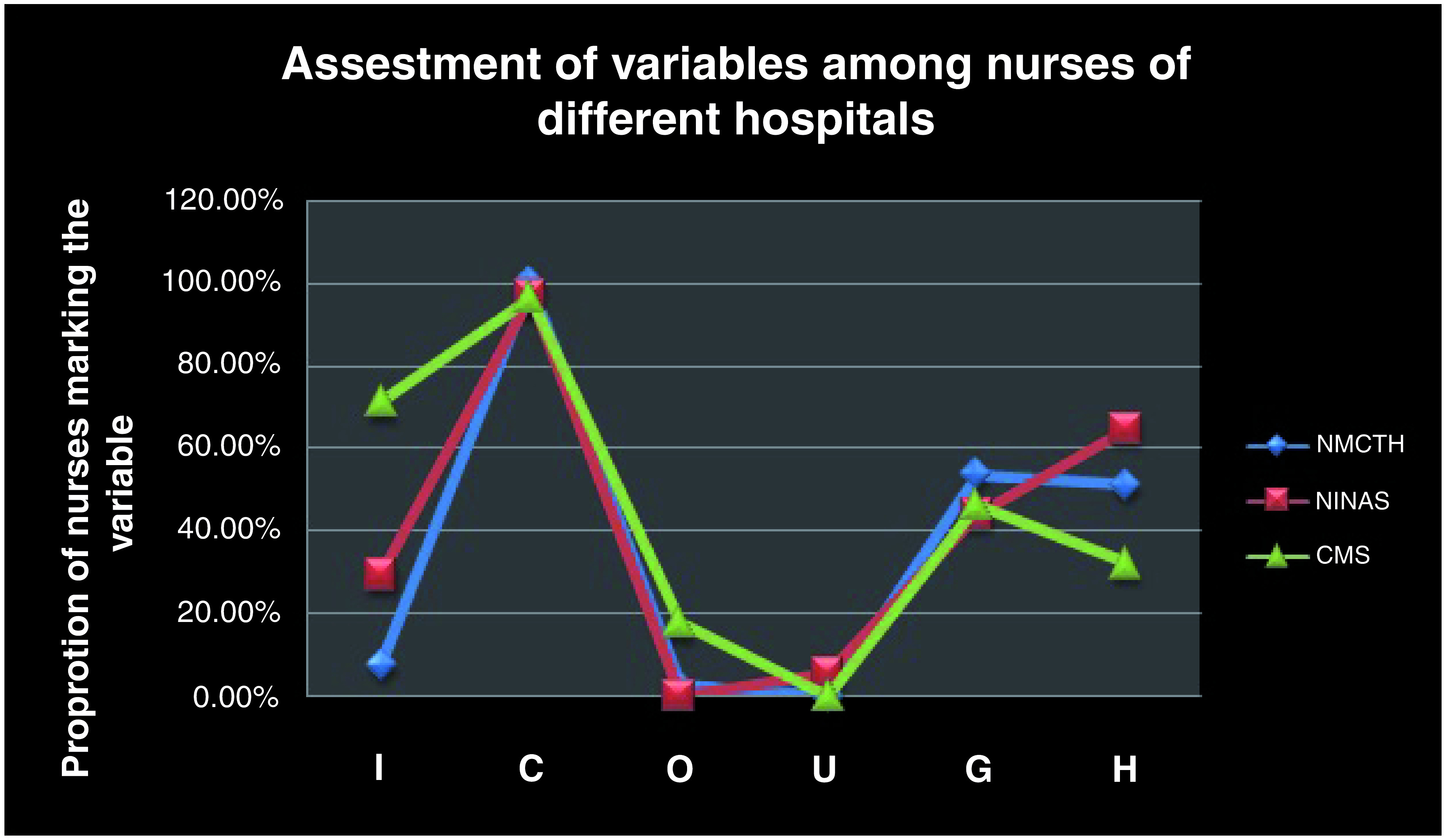
Assessment pertaining to different variables of I COUGH among nurses from all three hospitals. NMCTH - Nobel Medical College and Teaching Hospital, CMS - College of Medical Sciences, Chitwan, NINAS - National Institute of Neurosurgery and Allied Health Sciences.

In terms of individual variables of I COUGH, Cough and Head end elevations were mentioned by most number of nurses whereas Oral hygiene and Understanding aspects were mentioned by the least number of nurses ( Figure 2).

**Figure 3.**
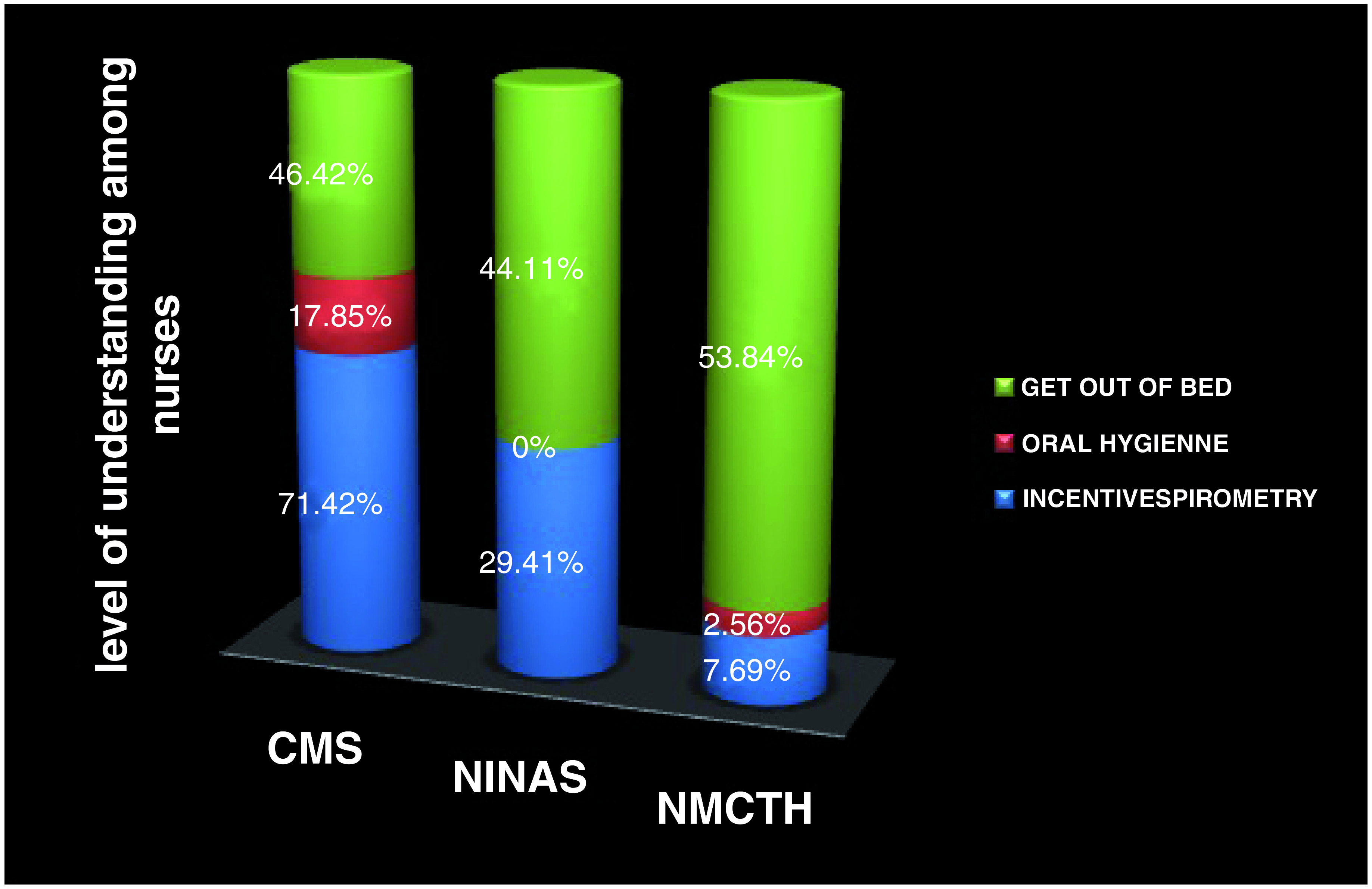
Level of understanding of individual variables within I COUGH. NMCTH - Nobel Medical College and Teaching Hospital, CMS - College of Medical Sciences, Chitwan, NINAS - National Institute of Neurosurgery and Allied Health Sciences.

**Figure 4.**
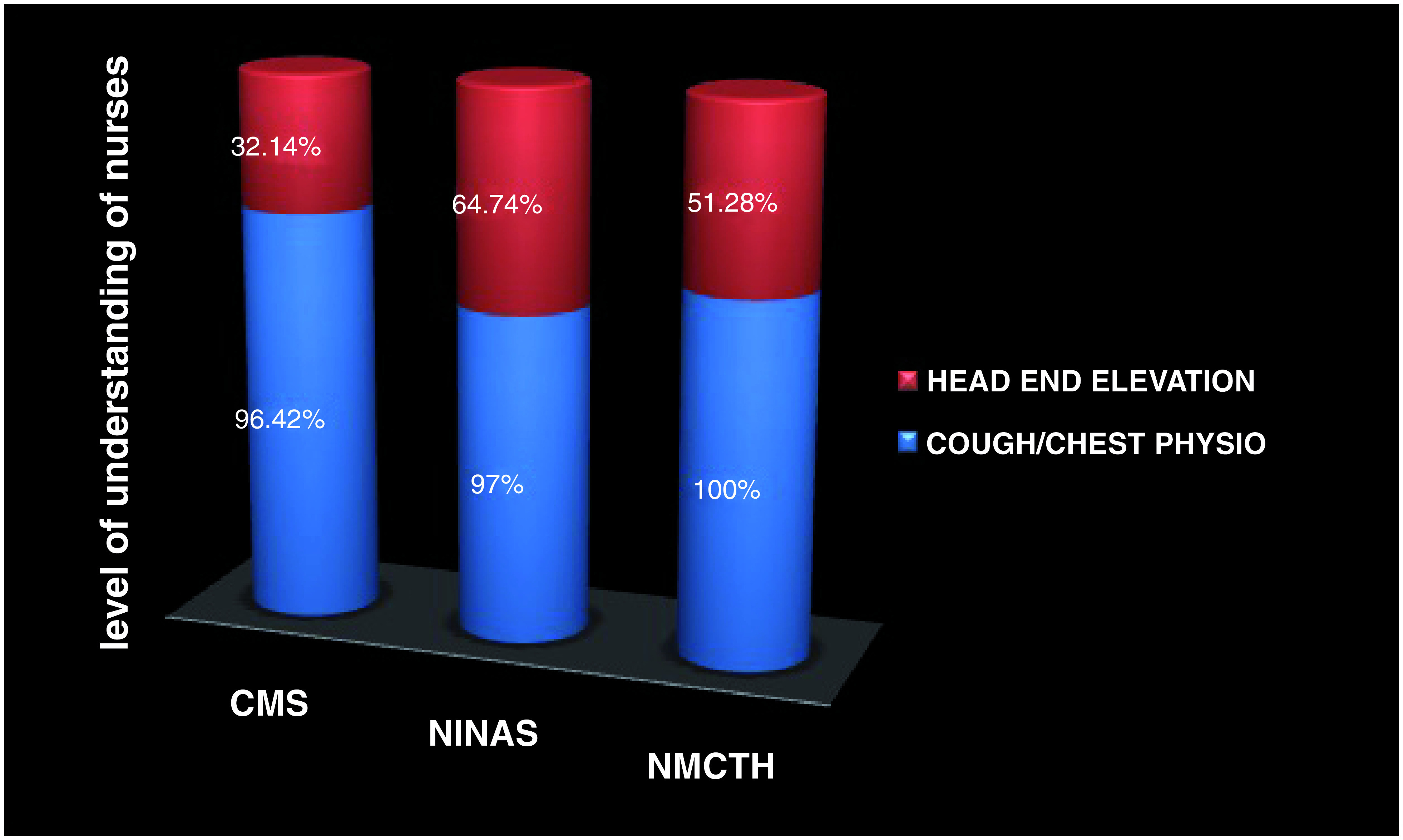
Level of understanding of individual variables within I COUGH. NMCTH - Nobel Medical College and Teaching Hospital, CMS - College of Medical Sciences, Chitwan, NINAS - National Institute of Neurosurgery and Allied Health Sciences.

Cough was mentioned the most (96.42%, 97% and 100% respectively) whereas oral hygiene was mentioned the least ( Figure 3 and Figure 4).

**Figure 5.**
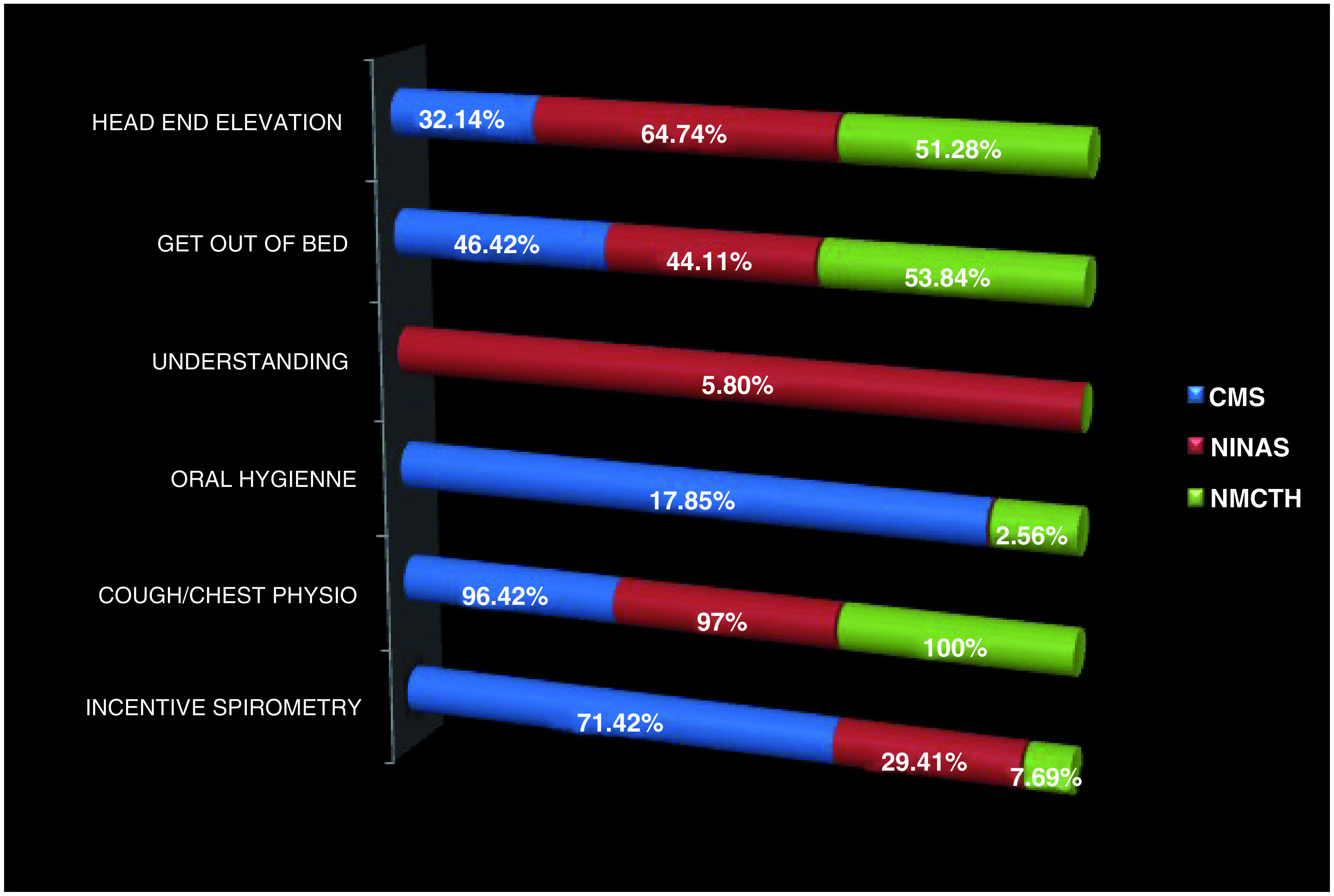
Assessment of all variables among nurses of all three hospitals. NMCTH - Nobel Medical College and Teaching Hospital, CMS - College of Medical Sciences, Chitwan, NINAS - National Institute of Neurosurgery and Allied Health Sciences.

In terms of individual variables, Incentive spirometry was mentioned by most nurses from College of Medical Sciences whereas Cough was mentioned the highest from nurses of Nobel Medical College and National institute of Neurosurgery and Allied Sciences institutions ( Figure 5).

Despite naming Cough correctly by most nurses from all three institutions, paradoxically none of the nurses correctly mentioned of all the essential components of Chest physiotherapy.

## Discussion

VAP in associated in 9–27% of all mechanically ventilated patients ^7^. One study showed incidence of 25 VAPs per 100 ventilated patients, thereby protracting ICU stays and increasing the risk of mortality among them ^8^. The provision of dedicated physiotherapists in critical care is still in its infancy in our context ^9^.

Nurses play pivotal role by standing in the frontline of the health surveillance systems and thereby are supposed to detect, make inferences and then implement the correct course of action during any adverse events in the ICU set up. This is undermined however, by the lack of trained staff. Moreover, there are frequent interruptions during in their assigned work schedule owing to factors such as unplanned emergency procedures, assisting for critical events, disjointed and missing medical supply etc. This increases the risks of some omissions in their care delivery amidst such stacking of cognitive and physical loads. However, merely increasing the number of nurses in a patient care unit may not be a panacea if the environment in which they work is not conducive to clinical decision making. There should be task organization skill simulations to improve their ability to manage such high cognitive managerial tasks ^10^.

The importance of effective clinical leadership in ensuring safe and efficient care has been reiterated time and again. Nurses need to gain autonomy over their own practicing behavior in order to improve their patients’ clinical outcome ^11^. Effective model study should be framed to upraise the standards of such pivotal role playing the front-line staffs ^12, 13^. Future shortages of nurses may be unpreventable, but making provisions for effective interdisciplinary teamwork, coupled with pivotal approaches for determining quality insurance and promoting safe environments for patients, could help mitigate their harmful impacts ^14^.

A similar study carried out to assess the knowledge of nurses regarding chest physiotherapy found that though they had good results in their clinical performances, with regards to clinical knowledge encompassing the same, they ironically performed poor ^15^. Almost 67% of them were not aware about indication of postural drainage while 76% were not aware about definition of percussion and vibration ^15^. Similar discrepancy with regards to task performance and the knowledge regarding underlying rationales for the same have been seen in our study as well. Our study also revealed that despite understanding that chest physiotherapy is an essential strategy to minimizing pulmonary complications, none of the nurses were able to correctly outline different components of the same namely positioning, percussion, vibration, squeezing and finally suctioning ^16, 17^.

Amidst the scenario of having an extreme shortage of effective manpower in the health sector on the one hand and the prevalence of huge discrepancy between knowledge of the tasks being performed among the healthcare practitioners on the other; the application of care bundle approaches could promote the notion of “effective tasking through minimal manpower”. This can help improve the standards of the health care providers as well as safeguard patient’s health and safety.

There are certain limitations to our study. Foremost being inclusion of nurses from Neurosurgical units of only three teaching hospitals of our country. One of the prevailing limiting factors in carrying out such observational study is the risk of coherent bias in the results due prior knowledge of such study among the probable study groups from their peers who already participated in the study. However, the results can further be validated through its multicentric application throughout national and international hospitals with inclusions of nurses from other surgical subspecialties as well as those practicing in the general incentive care units. There is also pivotal need in assessing the effect of such patient focused care bundle approaches in minimizing complications among the patients following its application. The fundamental rational of this study is to promote inclusions of patient care bundle approaches in managing patients to safeguard our patients’ health.

## Conclusion

The initiation of patient focused care bundle approaches is imperative to improving health delivery standards especially in low income nations. Such initiatives can promote health care provider autonomy on patient care, as well as assure better patient outcomes by minimizing complications.

## Data availability

### Underlying data

Open Science Framework: I COUGH PROJECT. https://doi.org/10.17605/OSF.IO/4U8C7 ^18^

This project contains the following underlying data:

- I COUGH DATA F1000.xlsx (Nurses scores for I COUGH and chest physiotherapy awareness)

### Extended data

Open Science Framework: I COUGH PROJECT. https://doi.org/10.17605/OSF.IO/4U8C7 ^18^

This project contains the following extended data:

- QUESTIONNAIRE.docx (Study questionnaire)

Data are available under the terms of the Creative Commons Zero "No rights reserved" data waiver (CC0 1.0 Public domain dedication).

## References

[1] Griffiths SV Conway DH POPC-CB Investigators: What are the optimum components in a care bundle aimed at reducing post-operative pulmonary complications in high-risk patients? *Perioper Med (Lond).* 2018;7:7. 10.1186/s13741-018-0084-9 29692886 PMC5904979

[2] Acharya SP: Critical care medicine in Nepal: where are we? *Int Health.* 2013;5(2):92–5. 10.1093/inthealth/iht010 24030108

[3] Murthy S Leligdowicz A Adhikari NK: Intensive care unit capacity in low-income countries: a systematic review. *PLoS One.* 2015;10(1):e0116949. 10.1371/journal.pone.0116949 25617837 PMC4305307

[4] Shrestha RR Vaidya PR Bajracharya GR: A survey of adult intensive care units in Kathmandu Valley. *Postgraduate Med J Nat Acad Med Sci.* 2011;11(2):1–7. Reference Source

[5] Paudel K: Report on status of nurses in Nepal. Nepal Health Research Council,2010;123456789 Reference Source

[6] Cassidy MR Rosenkranz P McCabe K: I COUGH: reducing postoperative pulmonary complications with a multidisciplinary patient care program. *JAMA Surg.* 2013;148(8):740–745. 10.1001/jamasurg.2013.358 23740240

[7] Kalanuria AA Ziai W Mirski M: Ventilator-associated pneumonia in the ICU. *Crit Care.* 2014;18(2):208. 10.1186/cc13775 25029020 PMC4056625

[8] Mishra DR Shah N Shah DS: Incidence and Outcome of Ventilator Associated Pneumonia in ICU of a Tertiary Care Hospital in Nepal. *JNMA J Nepal Med Assoc.* 2017;56(207):304–8. 10.31729/jnma.3216 29255310

[9] Baidya S Acharya RS Coppieters MW: Physiotherapy practice patterns in Intensive Care Units of Nepal: A multicenter survey. *Indian J Crit Care Med.* 2016;20(2):84–90. 10.4103/0972-5229.175939 27076708 PMC4810938

[10] Potter P Wolf L Boxerman S: An Analysis of Nurses' Cognitive Work: A New Perspective for Understanding Medical Errors.In: Henriksen K, Battles JB, Marks ES, *et al.*editors. *Advances in Patient Safety: From Research to Implementation.*(Research Findings). Rockville (MD): Agency for Healthcare Research and Quality (US);2005;1. 21249809

[11] Kieft RA de Brouwer BB Francke AL: How nurses and their work environment affect patient experiences of the quality of care: a qualitative study. *BMC Health Serv Res.* 2014;14:249. 10.1186/1472-6963-14-249 24923663 PMC4064111

[12] Needleman J Hassmiller S: The role of nurses in improving hospital quality and efficiency: real-world results. *Health Aff (Millwood).* 2009;28(4):w625–33. 10.1377/hlthaff.28.4.w625 19525289

[13] Clarke SP Donaldson NE: Nurse Staffing and Patient Care Quality and Safety.In: Hughes RG, editor. *Patient Safety and Quality: An Evidence-Based Handbook for Nurses*. Rockville, MD: Agency for Healthcare Research and Quality;2008. 21328775

[14] Buerhaus PI Donelan K Ulrich BT: Impact of the nurse shortage on hospital patient care: comparative perspectives. *Health Aff (Millwood).* 2007;26(3):853–862. 10.1377/hlthaff.26.3.853 17485766

[15] Kheder M: Assessment of Nurses Knowledge & Practice Regarding Chest Physiotherapy in Elmek Nimer University Hospital. master degree in medical surgical nursing. shendi university, faculty of post graduate studies and scientific research2016;28–30. Reference Source

[16] Van der Schans CP: Conventional chest physical therapy for obstructive lung disease. *Respir Care.* 2007;52(9):1198–206; discussion 1206–9. 17716386

[17] Ciesla ND: Chest physical therapy for patients in the intensive care unit. *Phys Ther.* 1996;76(6):609–625. 10.1093/ptj/76.6.609 8650276

[18] Munakomi S: I COUGH PROJECT.2019 10.17605/OSF.IO/4U8C7

