# Supplementary material for: A pilot study on assessing the gap between nurses’ task performances and knowledge pertaining the same with reference to “ I COUGH” initiative- a call for promoting patient ‘care bundle’ assignments in low-income nations
Source: F1000Res. 2019 Apr 23;8:531. [Version 1] doi: 10.12688/f1000research.18815.1 (PMC7359752; doi:10.12688/f1000research.18815.1)
Supplement: Peer review history

# Peer review: Reviewer response for version 1

**DOI:** 10.5256/f1000research.20615.r65132

**Published:** 2020-07-13

**Authors:** Nirmala Pradhan

**Affiliations:** 1 Department of Psychiatric Nursing, B.P. Koirala Institute of Health Sciences, Dharan, Nepal

1. Abstract: It is ok, but the result is focused only on knowledge.

 2. Methodology:

- How was the sample size (101) calculated? On what basis?
- Since the population of this study is Nurses, you need to define it.
- Did the nurses selected in all three settings have similar experience and educational level?
- Brief description of the knowledge questionnaire must be mentioned. How it was interpreted? Need to mentioned the reliability of the tool.

3. Result:

- Socio-demographic profile is not highlighted. Add on this.
- Figure 1 there is no index. Its not understandable [Color code used].
- If you have observed the Nurse’s task performance, nowhere its mentioned. It will be nice to include this aspect as your study is looking for practice gap. You can include like frequency of observation while caring the post-operative patients. Was it a participatory or non- participatory observation? How did you over-come the biases?
- Statistical analysis was extremely basic. Only descriptive analysis was used. You could have used inferential statistical analysis consulting the statistician.

4. Ethical consideration: How did you maintain the confidentiality and privacy of the nurses at the time of data collection?

 5. In your title you’ve mentioned that there is a gap in Practice and also mentioned to find the gap. I see that your second objective is un-answered. Please include it.

Is the work clearly and accurately presented and does it cite the current literature?

Yes

If applicable, is the statistical analysis and its interpretation appropriate?

No

Are all the source data underlying the results available to ensure full reproducibility?

Yes

Is the study design appropriate and is the work technically sound?

Partly

Are the conclusions drawn adequately supported by the results?

Yes

Are sufficient details of methods and analysis provided to allow replication by others?

No

Reviewer Expertise:

Psychiatric and Psychiatric Nursing

I confirm that I have read this submission and believe that I have an appropriate level of expertise to confirm that it is of an acceptable scientific standard, however I have significant reservations, as outlined above.

# Peer review: Reviewer response for version 1

**DOI:** 10.5256/f1000research.20615.r65133

**Published:** 2020-07-03

**Authors:** Valarmathi Selvaraj

**Affiliations:** 1 Gulf Medical University, Ajman, United Arab Emirates

Thanks for the opportunity to review the articles titled ‘A pilot study on assessing the gap between nurses’ task performances and knowledge pertaining the same with reference to “I COUGH” initiative- a call for promoting patient ‘care bundle’ assignments in low-income nations’. The concept behind this research is novel and impressive for me.

**Introduction:**

- There should be more info related to the background aspects of this research.
- The authors need to mention the pulmonary complication occurring after the surgery.
- Here the authors need to cite the articles related to ‘I COUGH’ programme.
- The authors need to explain about Care bundle approach method.     

**Methodology:**

- The I COUGH protocol has to be mentioned in detail mentioning all the six components to enable readers other than nurses to understand and interpret.  
- The study design needs to be explained under this section. What do the authors mean by ‘Analytical study?’. It needs explanation.
- The authors have mentioned 101 nurses were advised to tabulate different components of effective chest physiotherapy. The authors mentioned only about ‘C’ and the remaining other five components such as 'Oral care', 'Understanding', 'Get out of bed' and 'Head end elevation'.
- The methodology should be written in detail mentioning duration of study, how many samples the authors have collected in a day, and details on the short educational activity.
- Overall, it will be nice to divide the methodology into subsections to improve understanding.

**Results:**

- In the results part the authors have mentioned clearly about nurses knowledge but the authors never mention the knowledge of nurses in the methodology. This needs rewriting.
- Figure 3 and Figure 4 have the same headings and need to be modified.
- The authors need to perform inferential statistics with the results.
- In the results the authors have mentioned about level of understanding but in the discussion, in a few places the authors have mentioned ‘knowledge’. It is confusing and hence needs to be rewritten.

**Discussion:**

- In the first paragraph the authors did not mention what VAP is. This needs expansion and a probable explanation.
- The ‘task organization skill simulations’ need more elaborations.  

**Conclusion:**

- I would suggest the authors to mention about how to improve the health care standard. Conclusion should be written based on the objective. Hence it can be rewritten.

Is the work clearly and accurately presented and does it cite the current literature?

Partly

If applicable, is the statistical analysis and its interpretation appropriate?

I cannot comment. A qualified statistician is required.

Are all the source data underlying the results available to ensure full reproducibility?

Yes

Is the study design appropriate and is the work technically sound?

Partly

Are the conclusions drawn adequately supported by the results?

Yes

Are sufficient details of methods and analysis provided to allow replication by others?

Partly

Reviewer Expertise:

Nursing health education

I confirm that I have read this submission and believe that I have an appropriate level of expertise to confirm that it is of an acceptable scientific standard, however I have significant reservations, as outlined above.

# Peer review: Reviewer response for version 1

**DOI:** 10.5256/f1000research.20615.r65131

**Published:** 2020-07-01

**Authors:** Dibya Sharma

**Affiliations:** 1 School of Nursing, Manipal College of Medical Sciences, Pokhara, Nepal

**Introduction:**

- **1 ^st^ paragraph** – Citation number can be provided once at the end of the paragraph instead of repeating it a number of times. Similar correction can be done in 6 ^th^ and 7 ^th^ paragraph.

**Methods:**

- Is it a observational analytical or descriptive cross sectional study? I think the design adopted is descriptive cross sectional study.
- How did you do the scoring in percentage and average scoring (2.27, 2.45 & 2,54), please discuss about the scoring part in detail.
- **Data acquisition:** Is the data shown in the table? I think it’s better to write “frequency distribution is shown in the bar diagram and line graph."

**Results:**

- Please write the sample size n = 101 in all the figures.
- **Figure no. 2: **No need to keep percentage in the 2 decimal like 120.00%, 100.00%, 80.00% etc. instead it can be written as 120%, 100%, 80%, etc.
- I think there is repetition of the same data in line graph (Figure 2) and bar diagram (Figure 5)…can keep only of one those.

Is the work clearly and accurately presented and does it cite the current literature?

Yes

If applicable, is the statistical analysis and its interpretation appropriate?

Not applicable

Are all the source data underlying the results available to ensure full reproducibility?

Yes

Is the study design appropriate and is the work technically sound?

Partly

Are the conclusions drawn adequately supported by the results?

Yes

Are sufficient details of methods and analysis provided to allow replication by others?

Yes

Reviewer Expertise:

NA

I confirm that I have read this submission and believe that I have an appropriate level of expertise to confirm that it is of an acceptable scientific standard.

# Peer review: Reviewer response for version 1

**DOI:** 10.5256/f1000research.20615.r49482

**Published:** 2019-06-17

**Authors:** Enrique Castro-Sánchez

**Affiliations:** 1 NIHR Health Protection Research Unit in Healthcare Associated Infection and Antimicrobial Resistance, Imperial College London, London, UK

Dear authors,

 Many thanks for the opportunity to review your manuscript, reporting on the concordance between Nepali nurses’ knowledge and performance of clinical interventions included in the ICOUGH program targeting postoperative complications. The following comments and suggestions aim to clarify and improve the paper for the readers.

**Introduction**

 I thought that the content was meandering from one topic to other. For example, the first paragraph focused on the prevalence of surgical specialities, but then the following paragraph touched upon the Nepalese human resources crisis, particularly on nurses (I would suggest not to use ‘manpower’ and talk about ‘human resources’). I think that it would have been very useful to learn about the prevalence of surgical complications in Nepal or at least, the 3 hospitals sampled.

 Further, the ICOUGH program is mentioned, but would be useful to offer 1-2 sentences regarding any potential improvement obtained by ICOUGH. Additionally, I think that it would be necessary to expand a bit on each of the components, particularly what ‘oral care’, ‘understanding’ and ‘get out of bed’ entail. Finally, readers would benefit from any information about adaptation/cultural interpretation of the ICOUGH in Nepal.

 It would be necessary as well to offer some evidence about knowledge-performance gaps related to bundles, and even how effective these have been or not. There would be enough room to include such details with a revision of the content related to nursing workforce, which is the lengthiest in the Introduction.

**Methods**

 I could not really understand how the components of the ICOUGH initiative were corroborated in practice. The content mentions “We initially confirmed the practice of inclusions of all the variables listed within the I COUGH initiative among the daily assigned nursing task assignments in all these three hospitals”.

 Also, the authors talk about ‘short educational programme’, and it would be necessary to add further details about what the programme included. 

 There is a mention to Ethical considerations, but there is no information about the selection, recruitment, consenting process, as well as the number of nurses invited to take part that led to the 101 responses.

**Results**

 The authors could offer some further statistical measures, such as ranges, and if & are compared across centres, then it would be useful to consider whether to carry out a comparison of percentages analysis. Also, I feel that the text could include the % of the most frequently mentioned variables. 

 I suggest that the graphs avoid using 3D figures, which do not really offer much. Figure 1 should have axes and axes titles, and I am not sure about how well the figures facilitate comparison. Figure 2 should also include the missing axis title and remove the 120% if proportion of nurses. Each of the letters of ICOUGH should be defined.

 I suggest that Figure 3 removes the stacked results and presents 3 bars for each hospital, and same for Figure 5. This graph, additionally, seems not to have results for each of the hospitals…

**Discussion**

 The 1 ^st ^para introduces data unrelated to the study or the topic, by talking about physiotherapists. 

 The authors then shift the debate towards lack of staff but then also towards the need to ensure clinical leadership. I don’t understand the sentence “Nurses need to gain autonomy over their own practicing behavior in order to improve their patients’ clinical outcome” – what does “gaining autonomy” mean?

 The text is also confusing here: “There should be task organization skill simulations to improve their ability to manage such high cognitive managerial tasks” (what does “task organization skill simulations” mean?). Equally, this paragraph is not clear “Future shortages of nurses may be unpreventable, but making provisions for effec-tive interdisciplinary teamwork, coupled with pivotal approaches for determining quality insurance and promoting safe environments for patients, could help mitigate their harmful impacts.” Finally, the sentence “the application of care bundle approaches could promote the notion of “effective tasking through minimal manpower”. Needs referencing, and particularly the notion of “effective tasking”, particularly as task-based nursing is now and outdated approach to care.

**Conclusion**

 Could the authors offer some more evidence that “Such initiatives [ *patient focused care bundle approaches*] can promote health care provider autonomy on patient care”?

Is the work clearly and accurately presented and does it cite the current literature?

Partly

If applicable, is the statistical analysis and its interpretation appropriate?

Partly

Are all the source data underlying the results available to ensure full reproducibility?

Yes

Is the study design appropriate and is the work technically sound?

Partly

Are the conclusions drawn adequately supported by the results?

Partly

Are sufficient details of methods and analysis provided to allow replication by others?

Partly

Reviewer Expertise:

infection prevention and control, antimicrobial stewardship, health literacy, nursing

I confirm that I have read this submission and believe that I have an appropriate level of expertise to state that I do not consider it to be of an acceptable scientific standard, for reasons outlined above.
